# Precision Medicine in Peritoneal Dialysis: An Expert Opinion on the Application of the Sharesource Platform for the Remote Management of Patients

**DOI:** 10.3390/jpm14080807

**Published:** 2024-07-30

**Authors:** Loris Neri, Lorenzo Di Liberato, Gaetano Alfano, Valeria Allegrucci, Nicoletta Appio, Carla Bussi, Daniela Cecilia Cannarile, Ilaria De Palma, Silvio Di Stante, Rosa Pacifico, Vincenzo Panuccio, Silvia Porreca, Vincenzo Terlizzi, Silvia D’Alonzo, Giusto Viglino

**Affiliations:** 1 Nephrology and Dialysis Unit, Ospedale “Michele e Pietro Ferrero”, Via Tanaro, 7-9, Verduno, 12060 Cuneo, Italy; 2 Nephrology and Dialysis Unit, Department of Medicine, G. d’Annunzio University, Chieti-Pescara, SS. Annunziata Hospital, 66100 Chieti, Italy; 3 Nephrology Dialysis and Transplant Unit, University Hospital of Modena, 41124 Modena, Italy; 4 Nephrology Unit, Policlinico Gemelli of Roma, 00168 Roma, Italy; 5 Nephrology Operative Unit, ASST Spedali Civili Brescia, 25123 Brescia, Italy; 6 Nephrology, Dialysis, Hypertension Unit, IRCCS Azienda Ospedaliero-Universitaria of Bologna, 40138 Bologna, Italy; 7 Nephrology and Dialysis Unit, Policlinico Università A. Moro, 70124 Bari, Italy; 8 Department of Nephrology and Dialysis, Ospedale Santa Croce, Azienda Sanitaria Territoriale n 1, Pesaro-Urbino, 61121 Fano, Italy; 9 GOM “Bianchi-Melacrino-Morelli”, 89128 Reggio Calabria, Italy; 10 Telemedicine Referente ASLCN2—Nephrology and Dialysis Unit ASLCN2, Ospedale “Michele e Pietro Ferrero”, Via Tanaro, 7-9, Verduno, 12060 Cuneo, Italy

**Keywords:** automated peritoneal dialysis, cloud-based platform, remote monitoring, telemedicine

## Abstract

The management of end-stage kidney disease (ESKD) has been constantly evolving over the last decade with the development of targeted approaches. In this field, telemedicine and remote monitoring are based on the availability of new cyclers that allow for bidirectional communication (between patient and physician) and for the application of the Sharesource cloud-based platform. These technologies allow patients with ESKD to undergo automated peritoneal dialysis (APD) at home. However, these approaches are not well standardized and largely applied yet. Therefore, this study aimed to elaborate a protocol for the utilization of the Sharesource platform to facilitate the practical management of patients treated with APD. A series of expert meetings were held between September 2022 and January 2023 in Italy. The participants (ten nephrologists and five nurses) from nine Italian public dialysis centers shared their opinions, examined the current scientific literature in the field, and reviewed the key characteristics of the Sharesource system to achieve a common position on this topic. A detailed and practical document containing experts’ opinions and suggestions on the use of the Sharesource platform for the management of patients treated with APD was produced. This expert opinion might represent a new useful instrument in clinical practice for managing patients undergoing home-based peritoneal dialysis (PD) through the Sharesource platform, which is valid not only for Italy. These recommendations pave the way to novel patient-centered and personalized therapeutic approaches for ESKD and highlight the advantages of telemedicine and remote monitoring in the management of patients with ESKD undergoing PD and its positive impact on their quality of life.

## 1. Introduction

Peritoneal dialysis (PD) provides a safe and cost-effective kidney replacement therapy (KRT) for patients with end-stage kidney disease (ESKD) [1]. Automated peritoneal dialysis (APD), a modality of PD, has been evolving since the early 1960s as a revolutionary device for KRT, overcoming the limitations of continuous ambulatory PD (CAPD) [2]. In Italy, data from the recently published 8th National Census (Cs-22 of PD for the year 2022) showed that in centers that use PD (i.e., two-thirds of Italian public dialysis centers), PD was prescribed to 19.8% of incident patients and 14.9% of prevalent patients, with good outcomes in terms of duration and infective complications. Among prevalent cases, APD was the most frequent type of prescribed PD (56.6%) [3]. Telemedicine (TM) is particularly helpful for patients with ESKD undergoing PD [4,5,6] and its implementation in combination with remote monitoring (RM) in APD became possible in 2015 with a new cycler allowing for bidirectional communication between the patient’s home and the hospital team through a cloud-based platform (Sharesource (Baxter, Amia, Homechoice Claria, Kaguya, and Sharesource are trademarks of Baxter International Inc.), Baxter, Deerfield, IL, USA) [1,2]. Studies conducted during the COVID-19 pandemic demonstrated the role of RM in APD in such emergencies [7,8,9,10]. The International Society for Peritoneal Dialysis (ISPD) Guidelines recognize that RM is a fundamental tool for obtaining and optimizing a high-quality prescription that reduces the economic and social costs of dialysis treatment [11]. However, practical documents supporting clinicians in the daily management of these patients are still lacking. In this scenario, a scientific board was organized virtually to collect highly qualified opinions from nephrologists and nurses with specific expertise in the field of PD to develop a shared document with instructions for RM in PD.

Therefore, the objectives of the meetings were as follows:

(1) To develop an effective and comprehensive protocol for the utilization of the Sharesource platform, based on scientific evidence and good clinical practice;
(2) To facilitate the practical management of APD for patients with ESKD and to define the related benefits.

## 2. Materials and Methods

During several expert meetings, held between September 2022 and January 2023 in Rome, 15 Italian panelists (10 nephrologists and 5 PD nurses) from 9 centers with extensive expertise in PD (total number of patients on PD was 481, 67.2% of whom were on APD, as of 31 December 2022) and Sharesource (used in APD patients since 2016) shared their opinions about their clinical experience in the management of patients treated with APD through the Sharesource platform and reviewed its key characteristics. The methodology used was based on audit and group discussions. Each participant presented their experience. At the first meeting, the work was organized based on functions, effects, and final results. Separate groups analyzed these aspects and shared and re-discussed them with all participants until the final synthesis. Three in-person meetings and several videoconference meetings were necessary to complete the work. By examining the advantages of the platform along with the current clinical unmet needs, a common position on the application of Sharesource was achieved, and a detailed document containing practical recommendations was proposed as a clinical tool for healthcare professionals (HCPs) managing patients treated with APD.

## 3. Results

The experts identified three main topics to examine and include in the document: (1) the impact of Sharesource on the organization of resources; (2) the impact of Sharesource on dialysis prescription and small solute/urea clearance; (3) the impact of Sharesource on drop-out, quality of life (QoL), organization, and costs. Each topic addressed by the panelists was divided into sections and sub-sections.

### 3.1. The Impact of Sharesource on the Organization of Resources

#### 3.1.1. Definition of Exclusion Criteria for Patients from Using the Sharesource Platform

The Baxter APD device, integrated with a two-way remote patient management platform, requires that patients performing APD at home are connected via the internet to clinicians in kidney units [12]. The panelists agreed that treatment with PD at home through the Sharesource platform is always recommended to patients except for two sporadic situations in which Sharesource cannot be used:

▪ Absence of internet access at the patient’s home;
▪ Lack of patient consent to the use of the Sharesource platform.

#### 3.1.2. Management of Alerts

Alerts support HCPs in managing dialysis: using a color code flagging system, nephrologists can modify the operative parameters of the treatment remotely. Any issue is associated with a yellow (i.e., with priority) or red (i.e., with high priority) flag [13]. The dashboard should be reviewed by clinicians daily, and red flag alerts require prompt clinical evaluation and management [14]. The panelists agreed that nephrologists can set alerts even in the absence of clinically relevant issues, based on their own choice and prescription. They defined the types of APD alerts of the device, representing the most common APD-related practical issues (Table 1):

a. Lost treatment time

▪ Treatment time lost is between 15 and 30 min: a yellow flag appears.
▪ Treatment time lost is ≥ 30 min: a red flag appears.

This notice helps to detect reductions in the treatment time due to system errors or voluntary treatment interruptions.

b. Treatment modifications

▪ Lost dwell time: in the case of a modifiable dwell time modality, an alert reports the overall dwell time lost when inflow or outflow prolongs. This alarm is recommended for lost dwell time exceeding 30 min, with a yellow flag.
▪ Volume of therapy lost: an alert appears when the therapy volume is 10% lower than the threshold value of the total therapy volume set. This is a scarcely used alarm.
▪ Bypassed drainage: a notice, with a red flag, is displayed when one drain is bypassed. If one bypassed drain was established by the nephrologist, the alert is activated when two drains are bypassed.
▪ Change in initial drainage: a yellow flag appears if this change is above 50%. Nephrologists can set a higher percentage if variable initial drainage is expected or if low final volumes are set.
▪ Initial drainage bypass: a red flag is displayed when a drainage is bypassed at the beginning. Although this alert is not frequently used, it can be of help for patients with low volume of abdominal fluids to avoid pain during discharge.
▪ Device program change: although this alert is not frequently used, it can be helpful if the program stops.

c. Action control

▪ Excessive drainage: in case of drainage that is greater than expected, a red flag appears.

d. Patient intervention

▪ Bypass number of load/stop phase: a warning appears if the number of times the patient has performed a bypass during the fill or stop phase is two or higher, as set in the system. This alert is linked to a red flag.

e. System errors

▪ Events occurred during treatment: this alert is displayed after at least five events occurred during the treatment, regardless of the clinical relevance of these events. The recommended flag is red.

**Figure 1:**
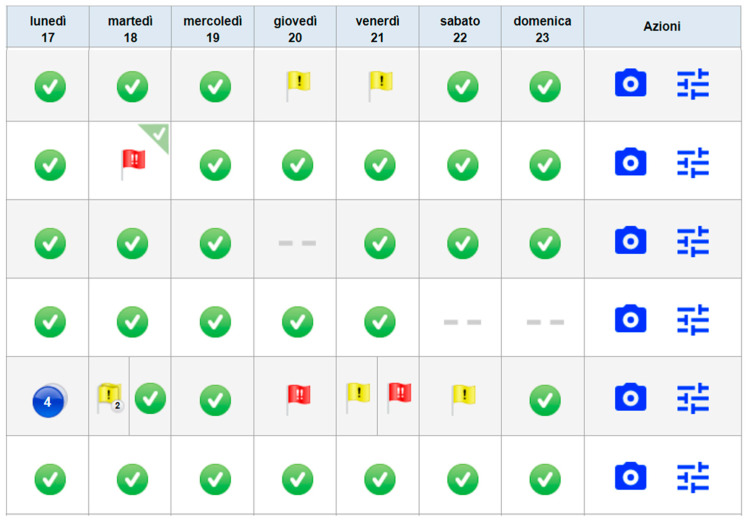
Sharesource dashboard displaying flag alerts. Red and yellow flags indicate the presence of issues, with the number of issues (if more than 2) displayed on them; green flags indicate a typical peritoneal dialysis course. Lunedì—Monday; Martedì—Tuesday; Mercoledì—Wednesday; Giovedì —Thursday; Venerdì—Friday; Sabato—Saturday; Domenica—Sunday; Azioni—Actions.

The dashboard where flag alerts, with different priorities (yellow or red flags), appear in case of issues, is depicted in Figure 1.

#### 3.1.3. Definition of Physician/Nurse Tasks

There is evidence that Sharesource APD may influence and modify the actions taken by physicians and nurses while managing patients. *Wood* and colleagues observed that time spent on proactive activities among nurses increased from 2% to 34% after the introduction of the cloud-based platform, with a reduction in routine and reactive tasks (e.g., assessing patients’ condition and history) [12]. An appropriate plan indicating the assignments of physicians or nurses for using the platform allows for the optimization of resources, which are scarce in most PD units. Therefore, the experts defined the specific tasks that physicians and nurses are in charge of in the management of patients undergoing APD with Sharesource.

a. Dashboard review

Nurses should be responsible for daily dashboard review and evaluation.

Red flags

▪ If any red flag alert is noticed, the nurse should immediately report it to the physician.

Yellow flags

▪ If a yellow flag is displayed for the first time, the nurse should review the overall data and graphs of the statistical trends in the treatment to identify possible causes. The nurse should verify and correct the problem, when possible, after talking to the patient and to the physician, if needed.
▪ If a yellow flag is displayed consecutively for two (or more) times, the nurse should contact the physician, since a prescription change could be necessary. In this contingency, the nephrologist can modify the treatment remotely or require an in-person visit before modifying the prescription.

b. Treatment data evaluation

During a monthly in-person visit, physicians should examine the report that contains the initial prescription, the pre-defined threshold parameters, and the real data. This report should also include the mean values of the parameters recorded over the period of interest (i.e., last 4 weeks). For patients with complicated conditions, data can be reviewed (totally or partially) daily or at any frequency considered appropriate by clinicians. Conditions that would require close and systematic medical evaluation are the following, according to the experts:

▪ First dialysis prescription;
▪ Severe comorbidities;
▪ Unexpected events occurring in a patient with a previously stable condition.

c. Patient/caregiver training for the use of Sharesource

The panelists highlighted the importance of explaining to patients and caregivers that RM does not replace in-person visits and that patients and caregivers should contact the PD center for any issue. The role of caregivers in assisting these patients undergoing PD (hence defined as “assisted PD”) is widely recognized [11], and in Italy, where in 2022, assisted PD was used in 21.1% of prevalent patients, caregivers are more commonly family members (86% according to the 8th GPDP-SIN census 2022) [3]. During the training of patients and caregivers, the role of communication between HCPs and patients should be emphasized. In most cases, the training is performed by nurses working at the PD center or by external specialized HCPs. In Italy, 58% of training courses occur in PD centers and 64% of them are held by an internal team of HCPs [15]. Panelists completely agreed about the need to evaluate whether patients and caregivers comprehended the instructions and their ability to use the system after the training. The main instructions for Sharesource platform use for patients and caregivers summarized by the experts included:

▪ Connecting the modem to the power supply;
▪ Inserting body weight and blood pressure data when required;
▪ Confirming treatment changes sent by the modem with possible phone support by HCPs.

#### 3.1.4. Procedure in Case of Technical Issues with the Sharesource Platform

The experts recommended asking for technical assistance from Baxter when technical issues occur, and summarized the most commonly reported issues as follows:

▪ Failure to send or receive data;
▪ Failure to register treatment data;
▪ Modem not working.

### 3.2. The Impact of Sharesource on Dialysis Prescription and Small Solute/Urea Clearance

#### 3.2.1. Treatment Coding

Dialysis solutions may differ based on the molecules that are contained in the solutions. Solution types commonly selected by HCPs include icodextrin (Extraneal: I), glucose-based PD solutions at variable concentrations, standard (lactate-based with acidic pH)- or low (bicarbonate-lactate-based with neutral pH)-glucose degradation product (GDP) solutions, and Aminoacid (Nutrineal: N) PD solutions. The choice of an adequate solution for PD is based on the patient’s characteristics. In order to have basic and concise information regarding the treatment for patients undergoing PD, the experts suggested inserting a 10-digit code in the field “Name of device program”, based on instructions specified by Table 2. Examples of 10-digit codes and their explanation in terms of solution types and contents are shown by Table 3.

#### 3.2.2. Optimization of Dialysis Prescription

The fill volume is defined based on several criteria: body size, intraperitoneal pressure, risk of hernia or other mechanical complications, and polycystic kidneys. Tidal peritoneal dialysis (TPD) is a modified PD described elsewhere [16] which is known to reduce pain during exchange and the number of night-time alarms [17]. The panelists agreed on preferring the TPD modality from the beginning, particularly if training is being carried out at home. They suggested starting treatment with a tidal volume of 50% and gradually increasing this percentage by 5–10%, based on established criteria:

▪ Low drain flow;
▪ Lost dwell time, for the modifiable dwell time modality;
▪ Prolonged treatment time, for the non-modifiable dwell time modality;
▪ Catheter functioning over different cycles;
▪ Pain perceived by the patient during the outflow phase.

If a tidal volume ≥80% is reached, a switch to a non-tidal APD is recommended since the non-tidal prescription is likely to improve dialysis efficiency and is easier to prescribe. If the training is carried out at the hospital or if it is known that drainage in the supine position of the catheter has a break point at >80% of the loaded volume, then it is possible to reverse the prescription procedure by starting with a non-tidal prescription and use tidal prescription only if flags arise, if the patient complains of abdominal discomfort, or if too much time is being lost. Nightly ultrafiltration (UF) in TPD can be prescribed by subtracting 200 mL from the mean nightly UF value measured by Sharesource in stable conditions. A further intermediate complete drainage might be added considering the criteria applied for the definition of tidal volume and UF volume. Sharesource allows for choosing between modifiable (fixed overall session duration; in case of increase or reduction in theoretical fill and drainage times, Home Choice Claria reduces or increases the duration of remaining stops) or non-modifiable dwell time (fixed dwell time; in case of increase or reduction in theoretical fill and drainage times, the overall duration of the treatment will increase or decrease accordingly) based on the medical prescription and the patient’s QoL.

### 3.3. The Impact of Sharesource on Drop-Out Rates, QoL, Organization, and Costs

**Figure 2:**
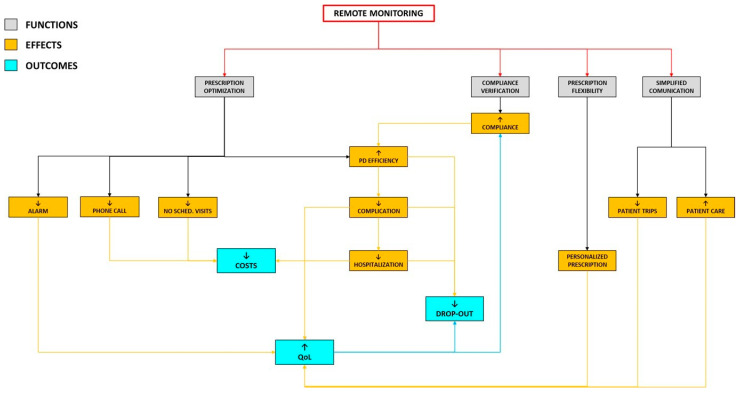
Relationship between the functions, effects, and outcomes of the Sharesource platform. Remote monitoring obtained by the functioning of the platform has direct effects on peritoneal dialysis course and on long-term outcomes. Abbreviations: PD, peritoneal dialysis; QoL, quality of life.

Figure 2 shows the relationship between the functions, effects, and outcomes of the Sharesource platform.

#### 3.3.1. Drop-Out Rates

Remote patient management (RPM) in PD can lessen drop-out rates by increasing patients’ self-confidence in home-based treatments [2]. The Cs-22 of peritoneal dialysis in Italy reported a global drop-out rate of 29.3 events/100 patient-year in 2022, primarily due to transfer to HD, death, and transplant. This rate remained substantially stable from 2005 to 2022, although a small increase has to be recognized (in 2016, a rate of 31.3 events/100 patient-year was recorded) [3]. The single main cause for transfer from PD to HD remains peritonitis (23.5% in 2022), but when considered together, UFF and insufficient solute (uremic toxins) removal represent the most frequent reasons for patient drop-out (29.1% in 2022) [3]. The panelists agreed on these reasons for drop-out reduction.

a. Remote management

According to the panel of experts, Sharesource allows for a reduction in PD drop-out rates thanks to the possibility of the remote monitoring of dialysis parameters and of remote prescription:

▪ Remote Monitoring:
  - Inflow and outflow time;
  - Observed UF as compared to expected UF;
  - Body weight and blood pressure;
  - Number and type of alerts;
  - Treatment adherence.
▪ Remote Prescription:
  - Quick adoption of remotely ordered prescriptions without traveling and with a reduced risk of complications.

b. Minimizing the most important reasons for patient drop-out

The possibility of monitoring dialysis parameters and prescription flexibility allows for a reduction in the following causes of patient drop-out [18,19]:

▪ Preventing insufficient clearance: based on lost dwell time, the number of alerts, and adherence to treatment, dialysis prescription can be modified to increase clearance in order to improve PD small solute/urea clearance (according to Section 3.2 of this guide).
▪ Preventing ultrafiltration failure (UFF): the adequacy of UF can be evaluated by recording it daily, weekly, and monthly and comparing to expected or prescribed UF based on body weight and blood pressure.
▪ Preventing catheter malfunction: by evaluating inflow and outflow times and alarm types, HCPs can detect the presence or the onset of catheter malfunction and properly modify prescriptions.
▪ Preventing poor compliance: by detecting recorded parameters, both prescribed and measured automatically by the device, HCPs can notice poor adherence in relationship to the following:
  - Skipped or reduced treatments;
  - Non-recorded body weight and blood pressure;
  - Procedure errors, such as non-authorized bypass;
  - Inability to manage alarms;
  - Non-reported events.

Depending on the type of issue, a re-training for patients/caregivers will be arranged or prescription will be changed.

▪ Burden of dialysis treatment by personalization of dialysis prescriptions that allows meeting the needs and life habits of patients and caregivers, after a deep and proper evaluation.

#### 3.3.2. Quality of Life

Recent studies assessing the QoL of patients through specific questions concerning PD home therapy monitoring indicate a greater acceptance of and satisfaction with care in patients monitored with RM than with standard PD [4,20]. The experts discussed how patients’ QoL may improve with Sharesource. They summarized the characteristics of the platform associated with a paramount impact on the overall QoL of patients treated with PD:

▪ Dialysis flexibility and personalization, depending on patients’ needs (e.g., preparation for surgery or diagnostic tests) and assistance (e.g., caregiver availability or different organization over the weekends);
▪ Better quality of sleep related to a reduction in the number of night alarms through the correct use of TPD;
▪ Reduction in complications and hospital admissions related to inappropriate dialysis (e.g., poor adherence to dialysis prescription; UF and/or depuration failure);
▪ Simplified communication between the patient/caregiver and HCPs in alarm management and dialysis prescription;
▪ Reduction in trips to dialysis units for both scheduled and non-scheduled visits (due to the need for medical evaluation or dialysis changes) with consequently reduced costs and increased time for work or hobbies;
▪ Confidence about continuous assistance and care, which increases patient self-confidence and minimizes worries about therapy;
▪ Better awareness of treatment and patient control in dialysis therapy.

#### 3.3.3. Organization and Costs

The advantages of RM in PD are well known. A retrospective cohort study on 360 patients treated with PD showed a significant reduction in hospitalization rate and hospitalization days when RM was applied to PD in comparison to PD without RM [1]. In 2018, a simulation study estimated a reduction in healthcare-associated costs with RM, mainly driven by avoided hospitalization, emergency visits, and unplanned clinic visits [21]. These results were recently confirmed by Amici et al., who reports how the use of Sharesource for six months is associated with a significant reduction in costs compared to the previous six months without Sharesource [3]. The experts agreed that a well-defined use of Sharesource may improve organization and reduce the center’s costs related to the optimization of dialysis prescription. In particular, they sustained that the non-stop monitoring of dialysis treatment ensured by Sharesource along with the proactive intervention can achieve the following:

▪ Reduce in-person non-scheduled visits to PD units;
▪ Reduce phone calls and emails aimed at change and management of dialysis prescriptions;
▪ Reduce complications related to inappropriate dialysis (e.g., poor adherence; UFF or clearance/adequacy failure);
▪ Reduce hospital admissions due to complications related to inappropriate dialysis (e.g., poor adherence to dialysis prescription; UFF and/or clearance/adequacy failure).

## 4. Discussion

In this document, Italian experts in the field of PD proposed detailed recommendations about the use of a new cycler that allows for bidirectional communication between patients’ homes and hospital teams through the cloud-based platform Sharesource. Even if some recommendations, particularly organizational ones, might seem country-specific, the document highlights the universal advantages of this modality of PD, in particular the decrease in patient drop-out and hospital visits. In addition, the panelists clarified how this approach may lead to the optimization of prescriptions and timely interventions. Patients undergoing home dialysis tend to be more engaged in their own treatment and experience a better QoL, with fewer drop-outs as a consequence. The effects of RPM, in particular when integrated with other forms of TM, allow for saving money and for a lower impact on ecology with PD by reducing the impact of dialysis on economic resources and patient QoL. Therefore, an awareness of the pivotal role of RPM in precision medicine for patients with ESKD undergoing PD led the panelists to compose a practical guide that physicians and nurses can follow and consult in their daily clinical practice when managing patients undergoing home-based PD through the Sharesource platform.

In conclusion, these recommendations highlight the current pivotal role of RPM in treating patients suffering from ESKD, paving the way to a patient-centered and personalized approach in the management of this clinical condition. This document spotlights the advantages of the recent advances in telemedicine as a tool to personalize the treatment of patients with chronic renal disease and provide novel solutions to old issues in the field of PD.

## Figures and Tables

**Table 1:** Alert types and associated flag colors.

Alert Types	Flag Color
LOST TREATMENT TIME	
15–30 min	Yellow
≥30 min	Red
TREATMENT MODIFICATIONS	
Lost dwell time	Yellow
Volume of therapy lost (>10%) *	N/A
Bypass drain > 2 times	Red
Change in initial drainage	Yellow
First bypass drainage	Red
Device program change *	N/A
ACTION CONTROL	
Excessive drainage	Red
PATIENT INTERVENTION	
Bypass number of load/stop phase	Red
SYSTEM ERRORS	
Events occurred during treatment ≥ 5	Red

* not frequently set, without an established flag color. Abbreviations: N/A, not available.

**Table 2:** Instructions for the 10-digit code for peritoneal dialysis treatment.

NIGHT EXCHANGE (fields 1–2–3–4)	
Y	1.36% glucose dialysis solution (yellow)
G	2.27% glucose dialysis solution (green)
R	3.86% glucose dialysis solution (red)
N	1.1% amino acid-based PD solution (nutrineal)
I	ICO-containing PD solution (extraneal)
DAY EXCHANGE (fields 5–6)	
Y	1.36% glucose dialysis solution (yellow)
G	2.27% glucose dialysis solution (green)
R	3.86% glucose dialysis solution (red)
N	1.1% amino acid-based PD solution (nutrineal)
I	ICO-containing PD solution (extraneal)
M	Mixed with glucose
ABDOMEN STATUS (field 7)	
E	Empty
F	Full
TIDAL VOLUME (fields 8–9)	
NUM	Tidal volume
DAYS/ALTERNATION (field 10)	
1–7	Number of days per week
A	Alternate day

Abbreviations: PD: peritoneal dialysis; ICO: icodextrin. The panelists suggested following specific instructions for entering treatment codes, which are as follows: (1) write in capital letters; (2) for fields “Bag 1–2–3–4”, insert glucose at increasing concentration, then amino acids (AAs) or the soluble glucose polymer ICO if they should be mixed with glucose; (3) if AA or ICO are not mixed with glucose, they should be inserted as the last infusion; (4) fill blank fields (if any) with an en dash.

**Table 3:** Examples of 10-digit codes for peritoneal dialytic solutions.

1	2	3	4	5	6	7	8	9	10
NIGHT EXCHANGE				DAY EXCHANGE		ABDOMEN	TIDAL %		DAYS
Heater bag	First bag	Second bag	Third bag	Last infusion	Second dwell	Initial abdomen status	Tidal percentage		Number/ Alternation
Y	G	R	-	I	-	F	7	5	5
G	Y	G	R	M	-	E	7	0	A
G	Y	G	-	N	M	F	6	5	3
Y	G	G	-	I	-	E	-	-	7

YGR-I-F755: solutions with increasing glucose concentration (from 1.36% to 3.86%) over the night exchange, then extraneal solution as last infusion during the day exchange, initial abdomen status: full, tidal volume 75%, 5 days per week. GYGR-E70A: solutions with different glucose concentration (up to 3.86%) over the night exchange, then a solution mixed with glucose, initial abdomen status: empty, tidal volume 70%, alternate days. GYG-NMF653: solutions with different glucose concentration (1.36% and 2.27% alternated) over the night exchange, then nutrineal solution as last infusion and during the day exchange mixed with glucose, initial abdomen status: full, tidal volume 65%, 3 days per week. YGG-I-V—7: solutions with increasing glucose concentration (from 1.36% to 2.27%) over the night exchange, extraneal solution as last infusion during the day exchange, initial abdomen status: empty, no tidal volume, 7 days per week.

## Data Availability

Not applicable.

## References

[1] Sanabria M., Buitrago G., Lindholm B., Vesga J., Nilsson L.-G., Yang D., Bunch A., Rivera A.S. (2019). Remote Patient Monitoring Program in Automated Peritoneal Dialysis: Impact on Hospitalizations. Perit. Dial. Int..

[2] Giuliani A., Crepaldi C., Manani S.M., Samoni S., Cannone M., De Cal M., Ronco C. (2019). Evolution of Automated Peritoneal Dialysis Machines. Contrib. Nephrol..

[3] Neri L., Viglino G., Vizzardi V., Porreca S., Mastropaolo C., Marinangeli G., Cabiddu G. (2023). Peritoneal Dialysis in Italy: The 8th GPDP-SIN census 2022. G. Ital. Nefrol..

[4] Manani S.M., Baretta M., Giuliani A., Virzì G.M., Martino F., Crepaldi C., Ronco C. (2020). Remote monitoring in peritoneal dialysis: Benefits on clinical outcomes and on quality of life. J. Nephrol..

[5] Neri L., Caria S., Cannas K., Scarpioni R., Manini A., Cadoni C., Malandra R., Ullo I., Rombolà G., Borzumati M. (2022). Peritoneal videodialysis: First Italian audit. G. Ital. Nefrol..

[6] Viglino G., Neri L., Barbieri S., Tortone C. (2020). Videodialysis: A pilot experience of telecare for assisted peritoneal dialysis. J. Nephrol..

[7] Ronco C., Manani S.M., Giuliani A., Tantillo I., Reis T., Brown E.A. (2020). Remote patient management of peritoneal dialysis during COVID-19 pandemic. Perit. Dial. Int..

[8] Polanco E., Aquey M., Collado J., Campos E., Guzman J., Cuevas-Budhart M.A., Divino-Filho J.C., Ramos-Sanchez A. (2021). A COVID-19 pandemic-specific, structured care process for peritoneal dialysis patients facilitated by telemedicine: Therapy continuity, prevention, and complications management. Ther. Apher. Dial..

[9] Alfano G., Fontana F., Ferrari A., Guaraldi G., Mussini C., Magistroni R., Cappelli G. (2020). Peritoneal dialysis in the time of coronavirus disease 2019. Clin. Kidney J..

[10] El Shamy O., Tran H., Sharma S., Ronco C., Narayanan M., Uribarri J. (2020). Telenephrology with Remote Peritoneal Dialysis Monitoring during Coronavirus Disease 19. Am. J. Nephrol..

[11] Brown E.A., Blake P.G., Boudville N., Davies S., de Arteaga J., Dong J., Finkelstein F., Foo M., Hurst H., Johnson D.W. (2020). International Society for Peritoneal Dialysis practice recommendations: Prescribing high-quality goal-directed peritoneal dialysis. Perit. Dial. Int..

[12] Wood E., McCarthy K., Roper M. (2019). Remote monitoring of peritoneal dialysis: Evaluating the impact of the Claria Sharesource system. J. Kidney Care.

[13] Crepaldi C., Giuliani A., Manani S.M., Marchionna N., Piasentin P., Ronco C. (2019). Remote Patient Management in Peritoneal Dialysis: Impact on Clinician’s Practice and Behavior. Contrib. Nephrol..

[14] Drepper V.J., Martin P.-Y., Chopard C.S., Sloand J.A. (2018). Remote Patient Management in Automated Peritoneal Dialysis: A Promising New Tool. Perit. Dial. Int..

[15] Neri L., Viglino G., Vizzardi V., Porreca S., Mastropaolo C., Marinangeli G., Cabiddu G. (2024). Peritoneal Dialysis in Italy: The 8th GPDP-SIN Census 2022—2nd Part: The Centers. G. Ital. Nefrol..

[16] Vychytil A., Hörl W.H. (2006). The role of tidal peritoneal dialysis in modern practice: A European perspective. Kidney Int..

[17] Neri L., Viglino G., Cappelletti A., Gandolfo C. (2001). Evaluation of drainage times and alarms with various automated peritoneal dialysis modalities. Adv. Perit. Dial..

[18] Corzo L., Wilkie M., Vesga J.I., Lindholm B., Buitrago G., Rivera A.S., Sanabria R.M. (2022). Technique failure in remote patient monitoring program in patients undergoing automated peritoneal dialysis: A retrospective cohort study. Perit. Dial. Int..

[19] Uchiyama K., Morimoto K., Washida N., Kusahana E., Nakayama T., Itoh T., Kasai T., Wakino S., Itoh H. (2022). Effects of a remote patient monitoring system for patients on automated peritoneal dialysis: A randomized crossover controlled trial. Int. Urol. Nephrol..

[20] Jung H.-Y., Jeon Y., Kim Y.S., Kim D.K., Lee J.P., Yang C.W., Ko E.J., Ryu D.-R., Kang S.-W., Park J.T. (2021). Outcomes of Remote Patient Monitoring for Automated Peritoneal Dialysis: A Randomized Controlled Trial. Nephron.

[21] Makhija D., Alscher M.D., Becker S., D’Alonzo S., Mehrotra R., Wong L., McLeod K., Danek J., Gellens M., Kudelka T. (2018). Remote Monitoring of Automated Peritoneal Dialysis Patients: Assessing Clinical and Economic Value. Telemed. J. e-Health.

